# Sweet’s Syndrome: A Classical Presentation of a Rare Disease

**DOI:** 10.1177/2324709619895164

**Published:** 2019-12-17

**Authors:** Arash Mollaeian, Hadi Roudsari, Ebrahim Talebi

**Affiliations:** 1 Medstar Health Internal Medicine Residency Program, Baltimore, MD, USA; 2 Nasseri Clinics of Arthritic and Rheumatic Diseases, Baltimore, MD, USA

**Keywords:** Sweet’s syndrome, neutrophilic dermatoses, immunology

## Abstract

Sweet’s syndrome, also known as acute febrile neutrophilic dermatosis, is a rare disorder that typically presents with rapid appearance of tender skin lesions accompanied by fever and leukocytosis with neutrophilia. Its pathogenesis is not fully understood. The syndrome is generally classified into classical, malignancy-associated, and drug-induced categories, each of which has its specific characteristics. In this article, we present a case of classical Sweet’s syndrome in a woman who presented with an acute viral illness.

## Introduction

Sweet’s syndrome, or acute febrile neutrophilic dermatosis, is a rare inflammatory condition that is characterized by appearance of abrupt painful papulonodular skin lesion in the setting of a prodrome of fever, leukocytosis with neutrophilia, and pathological findings of neutrophilic infiltration of the upper dermis in the absence of leukocytoclastic vasculitis. It is considered to be the major prototype of a subset of diseases known as neutrophilic dermatoses and is generally classified into 3 categories of classical (idiopathic), malignancy-associated, and drug-induced Sweet’s syndrome.^1-5^ The pathogenesis of Sweet’s syndrome remains unclear; however, the advances since its recognition have established the role of autoinflammatory processes involving both the innate and adaptive immune systems, eventually leading to their malfunction, resulting in immune-mediated hypersensitivity as well as involvement of cytokines such as interleukin-1β (IL-1β), IL-17, and tumor necrosis factor-α (TNF-α).^5-10^ A diagnostic approach using major and minor criteria is used globally to establish the diagnosis, and skin biopsy and finding of diffuse neutrophilic dermal aggregations in the absence of vasculitis has a pivotal role in making the diagnosis. Systemic corticosteroids remain the cornerstone of treatment strategies; however, other medications have been used as first line as well such as potassium iodide and colchicine.^5-9^

## Case Presentation

**Figure 1.**
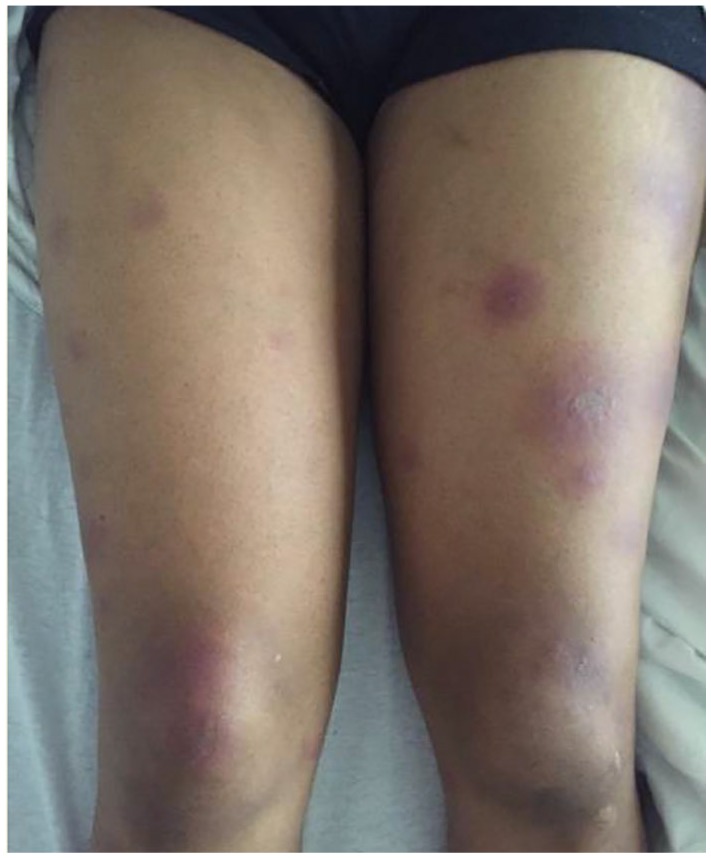
Patient’s lower extremity tender nodular rash on presentation.

**Figure 2.**
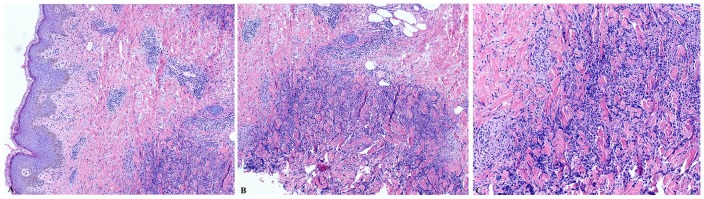
(A-C) Hematoxylin-eosin staining of the skin biopsy revealing papillary edema and dermal infiltration with neutrophils, evident on high- and low-power field microscopy.

A 41-year-old woman with past medical history of insomnia and anxiety presented with fever (as high as 103°F), sore throat, and generalized body pain for 6 days, accompanied by a painful rash involving lower extremities that later progressed to the trunk. During this period she visited the emergency department twice and was diagnosed with a flu-like illness and treated conservatively. However, her symptoms did not improve and she developed swelling of bilateral elbows, wrists, and metacarpophalangeals as well as a watery nonbloody diarrhea for 2 days before admission. On her third presentation to the emergency department she was febrile with a temperature of 39.8°C and appearing ill. She was noted to have symmetrical tender swelling of elbows, wrists, and metacarpophalangeals with decreased active and passive range of motion and dark erythematous, tender, nodular rash in bilateral thighs, abdomen, chest, and back (Figure 1). Her initial laboratory tests were significant for increased erythrocyte sedimentation rate to 85 mm/h and C-reactive protein to 131 mg/L without leukocytosis, neutrophilia, or bandemia. Blood cultures were drawn, and she was started on antibiotics and admitted to general medicine service. On evaluation by the primary team an extensive workup for infectious disease and basic rheumatologic screening was initiated. On consultation with infectious disease service, antibiotics were discontinued and skin biopsy was recommended, which was done the same day. The second day she remained febrile and continued to have the presenting symptoms especially the tender skin lesions without any improvement, and laboratory tests remained unremarkable without any leukocytosis or growth in cultures. Therefore, rheumatology service was consulted and she was started on pulse steroid therapy with 125 mg of intravenous methylprednisolone. On third day her fevers resolved and the rash and other symptoms started to rapidly improve. The next day, her infectious workup returned negative including HIV, monospot, influenza A and B, hepatitis C virus, hepatitis A virus, hepatitis B virus, chlamydia, and gonorrhea. Further the immunological workup revealed positive anti-neutrophil antibody of 1:80 (RO/SSA pattern); however, anti-neutrophil cytoplasmic antibody (myeloperoxidase and proteinase 3), C3, C4, anti-Ds-DNA, rheumatoid factor, anti-cyclic citrulinated peptide, RNP Ab, anti-cardiolipin Ab IgM/IgG (immunoglobulin) were negative or within normal limits (Table 1). She remained afebrile and her symptoms continued to improve and she was switched to 40 mg of PO prednisone daily and discharged on a prednisone taper. Later on her skin biopsy revealed dermal aggregates of neutrophils (Figure 2), and she was diagnosed with classical Sweet’s syndrome in the setting of a viral infection. She was evaluated by oncology and a full workup was unremarkable for any underlying malignancy. She also followed up with rheumatology as an outpatient and remained stable and symptom free.

**Table 1.** Laboratory Results of the Patient and Associated Normal Reference Range.

Laboratory Tests	Results	Laboratory Tests	Results
ESR	85 mm/h	HIV Ab/Ag	Nonreactive
CRP	131 mg/L	HIV DNA	Undetectable
ANA	Positive 1:80 (RO/SSA pattern)	Monospot	Negative
ANCA	Negative	Rapid flu A/B	Nonreactive
C3	176 mg/dL	HCV	Nonreactive
C4	35.8 mg/dL	HAV IgM	Nonreactive
Anti ds-DNA	Negative	HBC IgM	Nonreactive
RF	<10 IU/mL	HBs Ag	Nonreactive
Anti-CCP	12 U	*Chlamydia trachomatis*	Negative
RNP Ab	0 AU/mL	*Neisseria gonorrhoeae*	Negative
ACA IgG	<9 GPL	β-2-macroglobulin	2.6 HI
ACA IgM	11 MPL	β-2-glycoprotein I Ab (IgM, IgG, IgA)	WNL (2, 1, 4)

Abbreviations: ESR, erythrocyte sedimentation rate, HIV, human immunodeficiency virus; Ab, antibody; Ag, antigen; CRP, C-reactive protein; ANA, anti-neutrophil antibody; ANCA, anti-neutrophil cytoplasmic antibody; C3, complement 3; HCV, Hepatitis C virus; C4, complement 4; HAV, hepatitis A virus; Ig, immunoglobulin; ds-DNA, double-stranded DNA; HBC, hepatitis B core; RF, rheumatoid factor; HBs, hepatitis B surface; CCP, cyclic citrulinated peptide; ACA, anti-cardiolipin Ab.

## Discussion

Dr Robert Douglas Sweet first described and used the term acute febrile neutrophilic dermatosis, back in 1964 based on his observations in 8 women. However, the name “Sweet’s syndrome” is established as the eponym for acute febrile neutrophilic dermatosis and used worldwide. There is no specific racial or ethnic predominance for Sweet’s syndrome and it is distributed worldwide.^1-5^ Sweet’s syndrome is generally classified into 3 categories based on the underlying etiology and clinical scenario, including classical (idiopathic) Sweet’s syndrome, malignancy-associated Sweet’s syndrome, and drug-induced Sweet’s syndrome, all of which share the same presenting scenario of abrupt onset of tender papulonodular skin lesions, most commonly affecting the face, neck, and upper extremities with asymmetrical distribution, in the setting of fever and leukocytosis, with histopathologic findings of dense neutrophilic infiltration of the dermis without evidence of vasculitis.^1-5^ The classical Sweet’s syndrome is the most common type, which predominantly affects middle-aged women and is usually associated with an infectious process, usually involving the upper respiratory or gastrointestinal tract, inflammatory bowel disease, or pregnancy.^5-8^ Patients most commonly present with fever preceding the skin lesions that may be accompanied by general malaise, arthralgia, headache, and other symptoms such as flu-like illness, which rapidly respond to treatment with corticosteroids. These manifestations are very similar to those of familial Mediterranean fever; hence, there has been suggestion of possible common underlying pathophysiology.^6,10^ Symptoms may recur in up to one third of the patients, with or without treatment.^5-9^ Malignancy-associated Sweet’s syndrome was initially considered a subset of classical Sweet’s syndrome. The symptoms of Sweet’s syndrome can present concurrently, precede, or follow after the presentation of the associated malignancy. Skin lesion in cases associated with malignancy can be bullous or become ulcerated and resemble those of pyoderma gangrenosum.^5^ The most common malignancies associated with Sweet’s syndrome are hematological malignancies, most commonly acute myelogenous leukemia. Solid tumors with carcinomas of the genitourinary tract, breast, and gastrointestinal tract have been reported as well.^5-9^ Drug-induced form of Sweet’s syndrome is most commonly observed with granulocyte-colony stimulating factor, all-trans retinoic acid, trimethoprim-sulfamthoxazole, and azathioprine.^5,8^ The diagnostic criteria described by Walker and Cohen in 1996 relies on a temporal relation between administration of a specific medication and development of the specific symptoms as well as relapse of the symptoms with re-administration and resolution with discontinuation.^5-8^

Sweet’s syndrome is considered a subset of other neutrophilic dermatoses such as pyoderma gangrenosum and Behcet’s disease, all of which share the common pathophysiologic characteristics of autoinflammatory processes leading to neutrophilic infiltrations. The true pathogenesis of Sweet’s syndrome is unknown as of date and it is believed to be multifactorial and nonuniform between subtypes of the disease.^9-12^ Hypersensitivity is believed to be the underlying inciting mechanism driving the pathogenesis and the immune cascades leading to the disease manifestations; however, scarce evidence has been available to reveal the role of immune complexes and immunoglobulins, making the hypersensitivity hypothesis less strong. Photoinduction and Koebner phenomenon have also been suggested as possible inciting etiologies.^10^ Recent advances has led to the understanding that beside the innate immune system, the adaptive immune system also plays a significant role, evidenced by the elevated levels of IL-1α, IL-1β, IL-2, and interferon-γ, which are Type 1 helper T cells (Th1)-related cytokines. Further immunohistochemical examinations of the skin biopsies has revealed decreased Type 2 helper T cells (Th2), which indicates hyperexpression of Th1 cells and increased levels of TNF-α and interferon-γ, which lead to activation and recruitment of neutrophils. The role of proinflammatory T helper 17 (Th17) and secretion of IL-17 in neutrophil activation and recruitment as well as basement membrane remodeling have also been identified in the pathogenesis of Sweet’s syndrome. Moreover, recently there has been identification of certain genes and their roles in pathogenesis of neutrophilic dermatoses including Sweet’s syndrome. Human leukocyte antigen B54 has been associated with Sweet’s syndrome, as well as heterozygous mutations in MEFV gene that is observed in familial Mediterranean fever. These mutations seem to be activating the inflammasome and the innate immune system, leading to IL-1 production and neutrophilic cutaneous inflammation. Nevertheless, a unique pathway to the pathogenesis of Sweet’s syndrome remains to be elucidated.^9-12^ Diagnosis of Sweet’s syndrome was proposed by Su and Liu^3^ in 1986 and further modified by von den Driesch in 1994^4^ (Tables 2 and 3). It is based on clinical suspicion and mandates as skin biopsy, and exclusion of other potential differentials including infectious, inflammatory, and neoplastic processes.^3-10^

**Table 2.** Diagnostic Criteria for Classical and Malignancy-Induced Sweet Syndrome^a,5^.

*Major criteria:* 1. Abrupt onset of painful erythematous plaques or nodules. 2. Histopathology evidence of a dense neutrophilic infiltrate without evidence of leukocytoclastic vasculitis. *Minor criteria:* 1. Preceded by URT or GI infections or vaccinations; accompanied by a hematologic or visceral malignancies; inflammatory disorders; or pregnancy. 2. Pyrexia >38°C. 3. Leukocytosis >8000, neutrophilia >70%, ESR >20 mm/h, positive CRP (3 out of 4). 4. Excellent response to systemic corticosteroids or potassium iodide.

Abbreviations: URT, upper respiratory tract; GI, gastrointestinal; ESR, erythrocyte sedimentation rate; CRP, C-reactive protein.

a Two major criteria and 2 minor criteria are required to establish the diagnosis.

**Table 3.** Diagnostic Criteria for Drug-Induced Sweet’s Syndrome^a,5^.

1. Abrupt onset of painful erythematous plaques or nodules. 2. Histopathologic findings of dense neutrophilic infiltrate without evidence of leukocytoclastic vasculitis. 3. Pyrexia >38°C. 4. Temporal relationship between drug ingestion and clinical presentation, or temporally related recurrence after intake. 5. Temporally related resolution of lesions after discontinuation or treatment with systemic corticosteroids.

a All criteria are required to establish the diagnosis.

Management of Sweet’s syndrome lacks a universally accepted guideline; however, corticosteroids remain the cornerstone of first-line treatment, and an excellent response to steroids comprises one of the minor diagnostic criteria. Topical and intralesional steroids can be used for milder forms of the disease as well.^5^ Systemic steroids are usually used in doses of 0.5 to 1 mg/kg/day in both oral and intravenous forms, although higher doses up to 2 mg/kg/day have been used for more severe forms as well.^5-8^ Steroids are usually tapered in 3 to 5 days and when a desired clinical response is observed. Other commonly used first-line treatments consist of potassium iodide (900 mg/day) and colchicine (1.5 mg/day). Second-line treatments are also available such as clofazimine (100-200 mg/day), cyclosporine (2-4 mg/kg/day), dapsone (100-200 mg/day), and indomethacin (50-15 mg/day). Other less used medications such as antibiotics like doxycycline and metronidazole in cases of secondary infections, as well as other antimetabolite agents like cyclophosphamide, methotrexate, and anti-TNF agents such as etanercept and infliximab, have been reported in case reports and small series. Novel approaches to treatment have also been reported such as immunoglobulin and Anakinra (IL-1 receptor antagonist) in refractory cases.^5-9^ Efficacy of these treatment strategies and the variability in their mechanism of action, and advances in our understanding of neutrophilic dermatoses’ pathophysiology, especially TNF-α, IL-1β, and IL-17, indicates that both innate and adaptive immune systems play a pivotal role in pathogenesis of Sweet’s syndrome. Future research potential lies in further investigation of underlying immunologic signaling pathways, role of genetics in pathogenesis, associations and prognostic value in other autoimmune and myleoproliferative diseases.

## References

[1] Sweet RD. An acute febrile neutrophilic dermatosis. Br J Dermatol. 1964;76:349-356. doi:10.1111/j.1365-2133.1964.tb14541.x 14201182

[2] Sweet RD. Acute febrile neutrophilic dermatosis—1978. Br J Dermatol. 1979;100:93-99. doi:10.1111/j.1365-2133.1979.tb03573.x 427014

[3] Su WP Liu HN. Diagnostic criteria for Sweet’s syndrome. Cutis. 1986;37:167-174. 3514153

[4] von den Driesch P Sweet’s syndrome (acute febrile neutrophilic dermatosis). J Am Acad Dermatol. 1994;31:535-556. doi:10.1016/s0190-9622(94)70215-2 8089280

[5] Cohen PR. Sweet’s syndrome—a comprehensive review of an acute febrile neutrophilic dermatosis. Orphanet J Rare Dis. 2007;2:34. doi:10.1186/1750-1172-2-34 17655751 PMC1963326

[6] Anzalone CL Cohen PR. Acute febrile neutrophilic dermatosis (Sweet’s syndrome). Curr Opin Hematol. 2013;20:26-35. doi:10.1097/moh.0b013e32835ad132 23207661

[7] Rochet NM Chavan RN Cappel MA Wada DA Gibson LE. Sweet syndrome: clinical presentation, associations, and response to treatment in 77 patients. J Am Acad Dermatol. 2013;69:557-564. doi:10.1016/j.jaad.2013.06.023 23891394

[8] Villarreal-Villarreal C Ocampo-Candiani J Villarreal-Martínez A. Sweet syndrome: a review and update [in Spanish]. Actas Dermosifiliogr. 2016;107:369-378. doi:10.1016/j.ad.2015.12.001 26826881

[9] Marzano A Cugno M Trevisan V, et al Role of inflammatory cells, cytokines and matrix metalloproteinases in neutrophil-mediated skin diseases. Clin Exp Immunol. 2010;162:100-107. doi:10.1111/j.1365-2249.2010.04201.x 20636397 PMC2990935

[10] Heath M Ortega-Loayza A. Insights into the pathogenesis of Sweet’s syndrome. Front Immunol. 2019;10:414. doi:10.3389/fimmu.2019.00414 30930894 PMC6424218

[11] Marzano A Fanoni D Antiga E, et al Expression of cytokines, chemokines and other effector molecules in two prototypic autoinflammatory skin diseases, pyoderma gangrenosum and Sweet’s syndrome Clin Exp Immunol. 2014;178:48-56. doi:10.1111/cei.12394 PMC4360193 24903614

[12] Nelson C Stephen S Ashchyan H James W Micheletti R Rosenbach M. Neutrophilic dermatoses. Pathogenesis, Sweet syndrome, neutrophilic eccrine hidradenitis, and Behçet’s disease. J Am Acad Dermatol. 2018;79:987-1006. doi:10.1016/j.jaad.2017.11.064 29653210

